# Aging promotes neoplastic disease through effects on the tissue microenvironment

**DOI:** 10.18632/aging.101128

**Published:** 2016-12-06

**Authors:** Fabio Marongiu, Maria Paola Serra, Silvia Doratiotto, Marcella Sini, Maura Fanti, Erika Cadoni, Monica Serra, Ezio Laconi

**Affiliations:** ^1^ Department of Biomedical Sciences, Unit of Experimental Medicine, University of Cagliari, 09124, Cagliari, Italy

**Keywords:** aging, microenvironment, selection, pre-neoplastic hepatocytes, liver carcinogenesis

## Abstract

A better understanding of the complex relationship between aging and cancer will provide important tools for the prevention and treatment of neoplasia. In these studies, the hypothesis was tested that aging may fuel carcinogenesis via alterations imposed in the tissue microenvironment. Preneoplastic hepatocytes isolated from liver nodules were orthotopically injected into either young or old syngeneic rats and their fate was followed over time using the dipeptidyl-peptidase type IV (DPPIV) system to track donor-derived-cells. At 3 months post-Tx, the mean size of donor-derived clusters was 11±3 cells in young vs. 42±8 in old recipients. At 8 months post-Tx, no visible lesion were detected in any of 21 young recipients, while 17/18 animals transplanted at old age displayed hepatic nodules, including 7 large tumors. All tumors expressed the DPPIV marker enzyme, indicating that they originated from transplanted cells. Expression of senescence-associated β-galactosidase was common in liver of 18-month old animals, while it was a rare finding in young controls. Finally, both mRNA and IL6 protein were found to be increased in the liver of aged rats compared to young controls. These results are interpreted to indicate that the microenvironment of the aged liver promotes the growth of pre-neoplastic hepatocytes.

## INTRODUCTION

Aging is the strongest risk factor for neoplastic disease. However, the mechanistic links between these two complex biological processes are far from being fully elucidated. A time-dependent, progressive accumulation of critical mutagenic events in rare cells and/or failure to clear putative pre-neoplastic cells due to a decline in immune-surveillance are among the most considered hypothesis to relate aging and cancer [1-2]. On the other hand, there is now general consensus on the notion that the emergence of pre-neoplastic and neoplastic cell populations is heavily dependent on biological cues emanating from the host tissue microenvironment in which cancer arises [3,7]. Along these lines, we have observed that the imposition of a long-lasting constraint on the regenerative capacity of the liver is conducive to the rapid growth and progression of transplanted pre-neoplastic hepatocytes, while the same cells remain quiescent when injected into a normal, untreated host liver [8]. These results suggested that loss of regenerative potential in tissues where this property is functionally relevant may generate a biological driving force fostering the selective outgrowth of putative altered cells [4,9]. Since a reduced regenerative potential in several organs, including liver, is also a hallmark of aging [10,11], it is reasonable to hypothesize that alterations induced by the aging process might be similarly associated to the emergence of a neoplastic-prone tissue landscape, which would contribute to the increased frequency of cancer observed with advancing age [9]. Based on these premises, the present studies were undertaken to investigate the fate of pre-neoplastic hepatocytes isolated from rat liver nodules and orthotopically transplanted into syngeneic recipients of different age. The results indicate that the microenvironment of the aged host supports the growth and progression of pre-neoplastic hepatocytes; by contrast, no such expansion of transplanted cells was seen in the liver of young recipients.

## RESULTS

### Size of nodular cell clusters in young and old recipients

**Figure 1:**
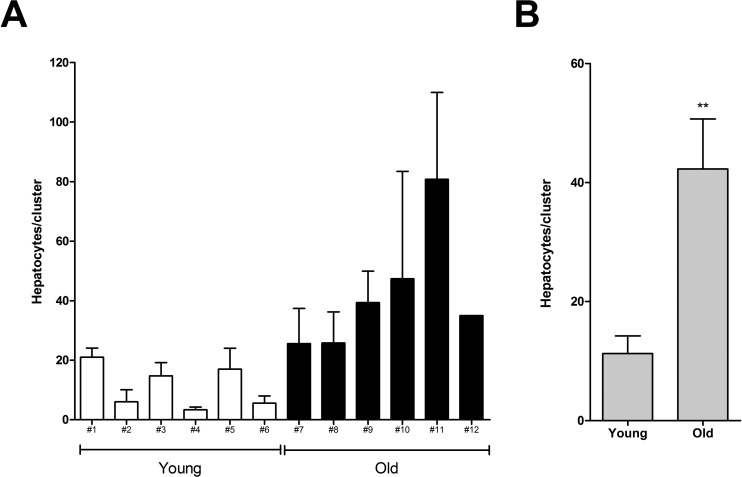
Size of donor-derived, pre-neoplastic hepatocyte clusters in animals transplanted at young or old age and killed 3 months after Tx (see Experimental procedures for details). Panel (A) mean cluster size (± SE) in individual animals. Panel (B) mean cluster size per group. Data are mean ± SE; **significantly different from young group:P<0.01

In a first study, we examined the size distribution of cell clusters originating from nodular hepatocytes trans-planted in either young or old recipient livers and analyzed at 3 months post-Tx. Panel A in Figure 1 shows the average number of cells per cluster in each animal. In rats transplanted at 4 months of age, the highest mean size of clusters per animal was 21±3, while the largest single cluster comprised 27 cells. By contrast, the same figures were much higher in the group transplanted at 18 months of age, being 81±29 and 563 cells, respectively. This finding was confirmed when the mean size of clusters was compared in the two groups (6 animals each, panel B in Figure 1): 11±3 cells per cluster were detected in young recipients, while this value was 42±8 in animals transplanted at 18 months of age.

### The growth of hepatocyte nodules in young and old recipients

**Figure 2:**
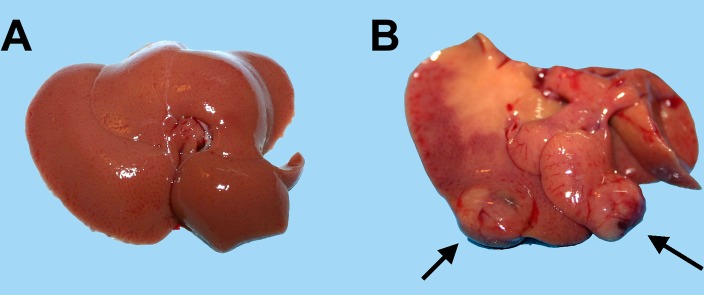
Macroscopic appearance of livers from animals transplanted with pre-neoplastic hepatocytes in at young or old age and killed 8 months after Tx. Panel (**A**) liver from a rat transplanted at 5 months of age and killed 8 months later: liver surface is regularly smooth and no lesions were detected. Panel (**B**) liver from a rat transplanted at 20 months of age and killed 8 months later: note the presence of two large nodules with prominent vasculature (arrows).

**Figure 3:**
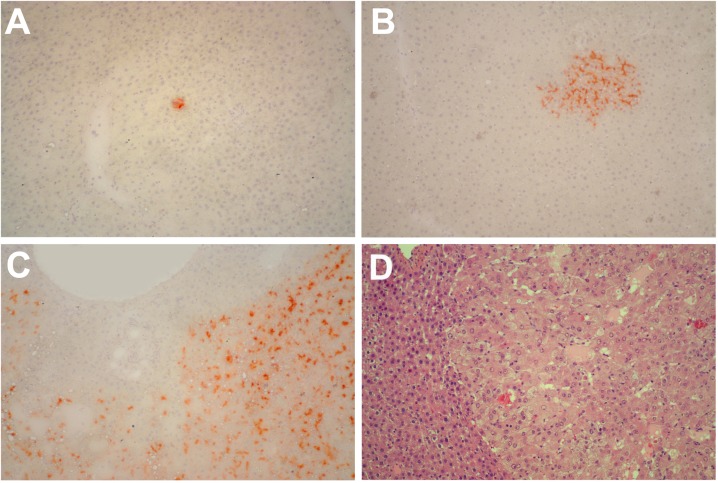
Panels (**A**) and (**B**) Histochemical staining for DPPIV of liver samples obtained from rats transplanted with pre-neoplastic hepatocytes at young age and killed 8 months after Tx. Small clusters of DPPIV-expressing hepatocytes (orange-rust) were discerned in all animals. Histochemical staining for DPPIV (panel **C**) and standard H&E staining (panel **D**) of a large hepatic nodule from a rat transplanted with pre-neoplastic hepatocytes at old age and killed 8 months later. Note the typical appearance of nodular hepatocytes arranged in multiple cell-thick plates. Magnification: 100x.

In a second series of experiments, young (3-5 months old) and aged (18-20 months old) Fischer rats were similarly transplanted with nodular hepatocytes and killed 8 months later. On macroscopic examination (Table 1), no visible lesion were seen in any of 21 recipient animals transplanted at young age (Figure 2, panel A). In stark contrast, 17/18 animals transplanted at old age displayed visible hepatic nodules, ranging from 1 to 8 mm in size, and including 7 larger tumors measuring up to 3 cm in diameter (Figure 2, panel B). All large lesions expressed the DPPIV marker enzyme, indicating that they originated from the transplanted cell population (Figure 3, panels A-C). Their gross appearance and structural morphology was similar to that of primary hepatocyte nodules generated through classical protocols for the induction of carcinogenesis in rat liver [16]. They were generally paler in color compared to surrounding tissue, displayed a prominent vasculature and were composed of two-three cell-thick hepatocyte plates (Figure 3, panel D).

**Table 1:** Incidence of hepatic lesions in animal transplanted with primary pre-neoplastic hepatocytes at young or old age

Age at Tx (months)	Animals with lesions	Total No. of Nodules (<1cm)	Size Range	Total No. of Tumors (>1cm)	Size Range
3-5	0/21	none	—	none	—
18-20	17/18	56	1-8 mm	7	1-3 cm

### Markers of cell senescence in aged rat liver

**Figure 4:**
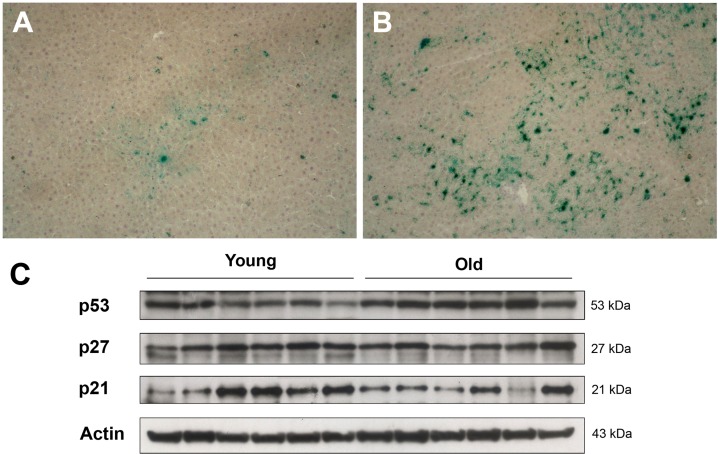
Panels (**A**) and (**B**) Expression of SA-β-gal in the liver of animals transplanted with pre-neoplastic hepatocytes in at young or old age and killed 3 months after Tx. The senescence marker was diffusely expressed in aged animals (panel **B**), while it was a rare finding in liver samples from young rats (panel **A**). Panel (**C**) Western Blot analysis of p21, p27 and p53 in liver samples from young and old recipients.

In order to identify alterations in the tissue microenvironment possibly relevant to the growth of transplanted cells in the aged liver, expression of markers of cell senescence was investigated. Cell senescence has long been interpreted as a fail-safe mechanism to limit neoplastic progression of altered cells [12]. On the other hand, it is now well established that it can also contribute to the emergence of the neoplastic phenotype, possibly through secretion of a host of factors, variably referred to as senescence-associated secretory phenotype (SASP) [13] or senescence-messaging secretome (SMS) [14], and comprising cytokines, growth factors and proteases. In light of the results reported above, expression of SA-β-gal, one of the most consistent markers of cell senescence, was determined in the liver of young vs. aged rats. Typical results are shown in Figure 4. Diffuse histochemical staining for SA-β-gal was observed in liver sections obtained from 18-month old animals, while it was a rare finding in the corresponding samples from young controls.

On the other hand, no significant changes were seen between young and aged animals in the hepatic expression of other markers that have been associated with cell senescence, such as the cell cycle inhibitors p21 and p27; however, aged livers displayed higher levels of p53, a central player in growth arrest following DNA damage [15].

**Figure 5:**
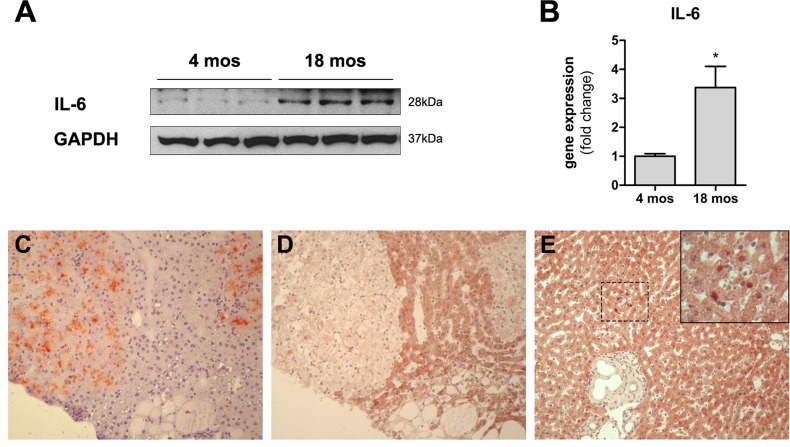
Panels (**A**) and (**B**) Expression of IL6 in the liver of animals transplanted with pre-neoplastic hepatocytes in at young or old age and killed 3 months after Tx. Both the protein (panel **A**) and the corresponding mRNA (panel B) were expressed at higher levels in the liver of aged animals. Panels (**C**) and (**D**) serial sections showing histochemical staining for DPPIV^+^ (panel **C**) and immuno-histochemical staining for STAT3 (panel **D**) in clusters of transplanted nodular hepatocytes. Only rare STAT3+ nuclei were detected in the aged rat liver (panel **E**). 100x magnification.

We next measured the levels of expression of IL6, a pro-inflammatory cytokine and a main component of SASP which has also been implicated in liver regeneration and repair [16]. Interestingly, both mRNA and IL6 protein were found to be increased in the liver of aged rats compared to young controls (Figure 5). In light of these results, we further investigated expression of the downstream effector of IL6, signal transducer and activator of transcription 3 (STAT3) in the liver of aged animals transplanted with nodular hepatocytes. However, only rare cells, scattered throughout the liver, showed nuclear staining for STAT3, with no specificity for clusters of donor-derived nodular cells.

## DISCUSSION

A better understanding of the bases of the intimate association between aging and cancer is likely to widen our opportunities to devise more effective approaches for the prevention and treatment of neoplastic disease. In order to unravel the intricacies of such complex relationship, biological coordinates need to be first firmly established. The results of these studies provide a contribution to this end, indicating that alterations in the tissue microenvironment of the aged rat liver can foster the growth of transplanted pre-neoplastic hepatocytes. They can therefore account, at least in part, for the increased risk of neoplastic disease associated with aging.

Primary pre-neoplastic hepatocytes were isolated from hepatic nodules generated according to a classical experimental model of chemical carcinogenesis [13,17]. They were then transferred into either young or old syngeneic hosts and their fate was followed over time for up to 8 months post-infusion, using the established DPPIV enzyme system as a tag [8,12].

The first finding to be highlighted is the very limited growth of transplanted cells seen in the liver of young recipients at either 3 or 8 months after Tx. Incidentally, we have now extended this observation to 12 months post-Tx, with similar results (data not presented). This indicates that the transplanted pre-neoplastic cell population is not endowed with any inherent degree of growth autonomy. Rather, its selective focal expansion appears to be heavily dependent on cues emanating from the tissue microenvironment, consistent with our earlier observations [8].

By contrast, the same cell population was able to grow and generate focal proliferative lesions, including large hepatocyte nodules, upon transplantation into the liver of aged animals, indicating that the microenvironment of the aged liver provides a promoting soil for the seeded pre-neoplastic cells isolated from a syngenic donor. In more general terms, these results support the conclusion that aging is associated with the emergence of a neoplastic-prone tissue landscape, which is likely to represent a key biological driving force to explain the increased incidence of cancer with advancing age. To our knowledge, this is the first study reporting on the fate of a primary pre-neoplastic cell population orthotopically transplanted in normal, untreated recipient animals of different age.

Our findings are reminiscent of those of McCullough et al. [17,18], who reported on the age-dependent regulation of the tumorigenic potential of neoplastically transformed rat liver epithelial cells by the liver microenvironment. However, those studies and ours differ in important experimental details, which impact on the interpretation and significance of results. Firstly, a rat liver epithelial cell line, grown *in vitro*, was employed by McCullough et al., while freshly isolated primary hepatocytes were used in the present investigation, with no *in vitro* passage. Secondly, the cell line in McCullough's studies was already neoplastic, with a full tumorigenic potential and a biological behaviour *in vivo*, which is difficult to predict and interpret. By contrast, cells isolated from hepatic nodules were pre-neoplastic, with no signs of growth autonomy. Furthermore, their biology has been extensively characterized: if left in the original host, they will progress to overt hepatocellular carcinoma in approximately 6 months from the time of isolation [19]. Overall, a continuity of the *in vivo* neoplastic process was maintained in the present experimental setup between the original host (the donor) and the recipient. Thus, our findings bear direct relevance to the pathogenesis of neoplastic disease as it occurs *in vivo* in experimental animals and in humans.

Important insights can be drawn from these results regarding the biological mechanisms mediating the effects of aging on carcinogenesis. A most entertained hypothesis places emphasis on the time-driven progressive accumulation of mutagenic events in rare cells that ultimately would lead to the acquisition of a neoplastic phenotype [1,20]. While our results are not at odds with this postulation, they do rule out the possibility that such mechanism is acting alone and/or it is of major biological relevance. In fact, the presence of altered, pre-neoplastic hepatocytes in the liver of young recipients does not result in any significant growth of focal lesions for several months post-transplantation, suggesting that it not sufficient, *per se*, to fuel carcinogenesis.

Another possibility that is often invoked to relate aging and cancer pertains to a gradual waning of the immune surveillance mechanisms in old age, such that altered/pre-neoplastic/neoplastic cells are no longer targeted for clearance and can therefore expand unchecked in the host [2,21,22]. Our present results would appear compatible with such scenario. However, we consider it unlikely for the following reasons. Firstly, donor and recipient animals are syngeneic and transplanted pre-neoplastic cells are still present in young host livers several months after transplantation, indicating that they are not cleared by the immune system. Secondly, we have obtained similar findings with normal hepatocyte transplantation [23], indicating that the microenvironment of the aged rat liver is more permissive for the growth of both pre-neoplastic and normal homotypic cells, which are not expected to be a target for clearance by the immune system in a syngeneic setting.

Over the last several years, cell senescence has taken centre stage for its possible involvement as a pro-carcinogenic stimulus. This concept has been specifically linked to the peculiar secretory phenotype of senescent cells, SASP, which includes cytokines, proteases and growth factors, among others, and can profoundly impact the biological response of the surrounding tissue [13,14,24-27]. Indeed, we found that incidence of cell senescence, as monitored by the expression of SA-β-gal, is increased in aged rat liver. Furthermore, levels of IL6, a pro-inflammatory cytokine and a major component of SASP which is also involved in liver regeneration and repair [16], were also higher in the liver of older animals. It is therefore reasonable to hypothesize that the presence of cell senescence and the accompanying SASP may mediate, at least in part, the observed promoting effect of the aged liver microenvironment on the growth of transplanted pre-neoplastic hepatocytes. Consistent with this postulation, we have previously shown that clearance of senescent cells delays carcinogenesis in a rat liver model [24].

However, other complementary mechanisms, such as cell competition [28-30], cannot be ruled out. The declining fitness of liver parenchyma with age, including a cell-autonomous decrease in proliferative potential [31], may well contribute to the selective emergence of altered cells[32], as it has been proposed in bone marrow during leukemogenesis [33,34,35]. Interestingly, it was recently reported that chronic inflammation associated with old age contributes to a reduced fitness of B cell progenitor populations, favoring the selection for cells harboring oncogenic mutations [36].

Irrespective of the specific mechanisms, our findings highlight an important role an age-associated altered tissue microenvironment in selecting for the emergence of pre-neoplastic cell populations. The finding of a direct pathogenetic link between aging and carcinogenesis reinforces the notion that similar strategies may help delaying both processes [37].

## MATERIALS AND METHODS

### Animals

All animals were maintained on daily cycles of alternating 12 h light/darkness with food and water available ad libitum. They were fed Purina Rodent Lab Chow diet (Ditta Mucedola, Italy) throughout the experiments and received humane care according to the criteria outlined in the National Institutes of Health Publication 86-23, revised 1985. Animal studies were reviewed and approved by the Institutional Animal Care and Use Committee of the University of Cagliari. In order to distinguish donor-derived from recipient cells in the liver, the dipeptidyl-peptidase type IV-deficient (DPPIV-) rat model was used [38]. A colony of DPPIV- F344 rats has been established in our laboratory, at the Department of Biomedical Sciences, University of Cagliari. DPPIV^−^ animals were used as recipients, while donor rats were syngeneic F344, DPPIV^+^ and were purchased from Charles River, Milan, Italy.

### Induction of liver nodules in donor rats and isolation of hepatocytes

Hepatocyte nodules were induced according to a well-characterized experimental model in the rat [19], as previously described [8]. Briefly, two-month old male Fischer 344 rats, expressing DPPIV enzyme activity, were injected with a single dose of diethylnitrosamine (DENA, 200 mg/kg. b.w., i.p., Sigma-Aldrich Chemical Co., St. Louis, MO) followed, 3 weeks later, by exposure to a modified version of the Solt and Farber protocol [39], to stimulate the growth of hepatocyte foci and nodules. Such protocol consisted of three consecutive daily doses of 2-acetylaminofluorene (20 mg/kg b.w., given by gavage tube, from Sigma-Aldrich) followed, on the fourth day, by a single administration of CCl_4_ (0.2 ml/kg b.w., by gavage, mixed in corn oil, 1:1 v:v). Six months after the initial treatment livers were perfused according to a standard 2-step collagenase perfusion technique [40,41]. Typically, 3 to 5 large (5-10mm in size) persistent nodules are present in the liver at this time point using the above experimental protocol. When left in situ, a subgroup of these nodules (an average of 1 or 2 per animal), will progress to cancer within about 1 year [19]. Large (>5 mm) nodules were physically separated from surrounding tissue and isolated cells were suspended in PBS and prepared for transplantation experiments. Prior to transplantation, cell suspension was filtered through a nylon mesh with a pore diameter of 100μ, in order to eliminate any large cell clumps. Cell viability, determined by trypan blue dye exclusion, was ∼85% in the nodular cell preparation.

### Hepatocyte transplantation

Young (3-5 months old) or aged (18-20 months old) Fisher 344 rats of the DPPIV^−^ strain were used as recipients. They were injected with 5×10^5^ cells freshly isolated from DPPIV^+^ hepatocyte nodules, via a mesenteric vein [8]. Animals from various groups were sacrificed at different time points during the experiment, as indicated in the Results section. Liver samples were fixed in 10% buffered formaldehyde or snap frozen. Histochemical determination of DPPIV enzyme activity was performed as described [8]. At least ten random sections were cut from each liver lobe of each animal and stained for DPPIV enzyme activity. Sections were then analyzed under the microscope and the number of hepatocytes for each DPPIV+ cluster was evaluated. Data from two separate experiments are reported. Data represent means±S.E. Statistical analysis was performed using the Student t test.

### Staining for senescence associated β-galactosidase activity

Detection of senescence associated β-galactosidase (SA-β-Gal) activity was performed as described [42]. Immediately before staining, X-Gal stock solution was prepared by dissolving 20mg/ml X-Gal (Invitrogen, Carlsbed, CA) in dimetylformamide. SA-β-Gal staining solution was prepared as follows: 1 mg/ml of X-Gal stock solution were dissolved in 40 mM citric acid in sodium phosphate, pH 6.0/5 mM potassium ferrocyanide/5 mM potassium ferricyanide/150 mM NaCl/2 mM MgCl2. Frozen sections of 10-μm thickness were fixed for 5′ in 4% formaldehyde/0.5% glutaraldehyde at 4°C, washed in PBS and incubated in fresh SA-β-Gal staining solution for 16h at 37°C. No special blocking step is required to perform the staining. Sections were counterstained with Hematoxylin.

### Histochemistry and immunohistochemistry

To follow the fate of transplanted cells, histochemical detection of DPP-IV positive clusters was performed on 5μm frozen sections as previously described [38].

Immunohistochemical staining for p-STAT 3 was performed on serial frozen sections. Slides were fixed with 3% formalin, blocked for 30′ and incubated with the primary antibody (Cell Signalling Technology, Danvers, MA) overnight at 4°C. Detection of specific signal was accomplished using an HRP/AEC detection IHC Kit (Abcam, Cambridge, UK).

### Western blot

Liver tissue samples were homogenized in RIPA lysis buffer containing Protease Inhibitors, then centrifuged at 12000 rpm for 30′ at 4°C. Protein concentration in supernatants was measured using the BCA method [42]. Samples (20μg protein) were prepared in Laemmli buffer, boiled at 95°C for 5′ then loaded into SDS-PAGE precast gels (Biorad, Hercules, CA) and run under denaturing conditions. Proteins were transferred onto nitrocellulose membranes (Amersham, UK), blocked with 5% non-fat milk for 1 h, then incubated with primary antibody for p21, p27 and p53 (Santa Cruz, Santa Cruz, CA), IL6, beta actin and gapdh (Abcam) overnight at 4°C. Membranes were washed and incubated for 2 h with the appropriate secondary antibody conjugated with HRP. Protein bands were detected using a chemoluminescent substrate (Biorad) and imaged onto Kodak film.

### RNA isolation, RT-PCR and Real-Time qPCR

Total RNA was isolated using TRIzol reagent (Invitrogen, Carlsbad, CA) according to the manufacturer's protocol. RNA integrity and purity were confirmed by 1% agarose gel electrophoresis and OD260/OD280 nm absorption ratio >1.8. Two grams of DNase-I treated RNA of each sample were reverse-transcribed by PCR using Promega reagents. The resulting cDNA was analyzed by quantitative real-time PCR using specific TaqMan assays and TaqMan Gene Expression Master Mix on an StepOne System (all from Applied Biosystems, Carlbad, CA). The rat specific assays were: IL-6 (Rn01410330_m1); β2-microglobulin (Rn00560865_m1). For both assays the thermal profile was as follows: 50˚C for 2 minutes, 95˚C for 10 minutes, 45 cycles at 95˚C for 15 seconds and 60˚C for 1 minute. Fold change was calculated by the 2-ΔΔCT method [43].
